# The effect of exercise on walking economy in patients with chronic neurological conditions: A systematic review and meta-analysis

**DOI:** 10.3389/fneur.2022.1074521

**Published:** 2023-01-11

**Authors:** Bowen Liu, Jingxuan Yu, Qiwei Fan, Fengwei Hao, Jinlong Wu, Wen Xiao, Fengyu Yu, Zhanbing Ren

**Affiliations:** ^1^College of Physical Education, Shenzhen University, Shenzhen, China; ^2^Department of Sports Medicine and Rehabilitation, Peking University Shenzhen Hospital, Shenzhen, China; ^3^School of Physical Education and Sports Exercise, South China Normal University, Guangzhou, China; ^4^College of Physical Education, Southwest University, Chongqing, China

**Keywords:** exercise, walking, energy cost, chronic neurological conditions, rehabilitation

## Abstract

**Introduction:**

To investigate the effect of exercise on the walking economy (WE) of patients with chronic neurological conditions (CNCs) and to determine the type of physical activity that best improves the WE of patients with CNCs.

**Methods:**

Four electronic databases were searched until December 2022 (Web of Science, PubMed, Cochrane, and CINAHL). Studies were screened using the following inclusion criteria: 1. randomized controlled or non-randomized controlled trials; 2. exercise interventions >4 weeks in duration; 3. patients aged ≥18 years with a diagnosis of CNCs. 4. walking economy of patients measured before and after the intervention. The PEDro scale was used to assess the methodological quality of the included studies.

**Results and discussion:**

Twenty-two studies met the inclusion criteria. Meta-analysis results showed that exercise significantly improved WE (g = −0.352, 95% CI, −0.625 to −0.078, *P* = 0.012). Subgroup analysis revealed that patients who received exercise showed better WE compared with those who underwent no control intervention (g = −0.474, 95% CI, −0.636 to −0.311, *P* < 0.001). However, exercise therapy did not show a significant improvement of WE compared with control groups (g = −0.192, 95% CI, −0.451 to 0.067, *P* = 0.146). In addition, we found that endurance combined with resistance, high-intensity intermittent, and other training modalities resulted in better WE compared with the pre-intervention. Of these, interval training has the greatest effect on improving WE. In conclusion, exercise can improve WE in patients with CNCs. More randomized controlled trials are necessary for the future.

**Systematic review registration:**

https://www.crd.york.ac.uk/prospero/display_record.php?ID=CRD42022361455, identifier: CRD42022361455.

## 1. Introduction

Globally, approximately 1 billion people suffer from chronic neurological conditions (CNCs), which have been becoming the main cause of death and disability in the world (1). Epidemiological studies have shown that the prevalence of CNCs has continued to increase over the years (2). The ability to exercise is considered an essential challenge in patients with CNCs. Lower exercise ability usually leads to health deterioration and worse quality of life for patients with CNCs (3–6).

For patients with CNCs, the intervention may be lifelong (7). In some patients, internal surgery is expensive and risky, and the use of other medications is associated with side effects and some of them do not efficiently restore body functions and improve daily activities (8). Therefore, exercise training is increasingly being used in the field of rehabilitation of exercise ability for patients with CNCs. It can improve various exercise functions such as balance, walking performance, and gait parameters in patients with CNCs such as stroke, Parkinson's disease (PD), and multiple sclerosis (MS) (9–14). Exercise for patients with CNCs can be broadly classified as endurance training (ET), resistance training (RT), endurance combined with resistance training (ERT), intermittent training (IT), or other training modalities (OTM). OTM is used to target specific functional impairments, for example, using the treadmill or ground-based walking exercises to improve walking ability in patients with stroke (15). Compared with usual care, exercise training exists for more intense physical activity and, more significantly, they are more economical and lifelong participation. A recent meta-analysis showed that exercise improved motor participation in patients with multiple sclerosis (15). It can, therefore, provide sustainable patient recovery.

Walking economy (WE) was defined as the steady-state aerobic demand at a given submaximal speed or distance, which usually was used to measure the energy cost while walking (16–18). A higher WE indicates that a patient can walk further per unit of time and distance. WE is influenced by age, and a meta-analysis reported a 17% increase in net metabolic cost in older adults compared with healthy younger adults (19). Disorders caused by neurological disorders increase energy expenditure during walking (16, 20, 21), making patients more prone to fatigue when walking (16, 22). Patients with stroke (22), PD (16), Alzheimer's disease (AD) (23), MS (24), and spinal cord injury (SCI) (25) exhibit higher oxygen consumption compared with healthy individuals. The mechanisms underlying the high energy cost in patients with CNCs may include tremors (16), walking biomechanics (26), and neural mechanisms (27). A poor WE may increase the risk of fatigue in patients with CNCs, which in turn causes functional limitations and reduces their quality of life and social participation (28–30). Therefore, strategies meant to improve WE have been explored to improve the recovery of motor ability in patients. However, to date, no high-quality studies have systematically reviewed the effects of exercise on WE. Several studies have explored whether exercise therapy can improve cardiopulmonary function in patients with CNCs. However, such studies did not test the value of peak oxygen consumption (VO_2peak_) improvement as a potential physiological indicator of cardiopulmonary function (10, 12, 14, 31–33), and this aspect has not been sufficiently reviewed as a primary outcome.

Given the importance of WE in the daily life of patients with CNCs, we think a review and analysis of the current literature is necessary. Therefore, the main objectives of this meta-analysis were (1) to assess the impact of using exercise on WE in patients with CNCs and (2) to different exercise modalities in an attempt to find an intervention that improves WE optimally.

## 2. Methods

The meta-analysis is based on the Preferred Reporting Items for Systematic Reviews and Meta-Analyses (PRISMA). The systematic evaluation program is registered in PROSPERO (CRD42022361455).

### 2.1. Literature search

A search was performed on the following electronic databases: PubMed, Web of Science, Cochrane, and CINAHL. The search was conducted from the earliest record to December 2022 using the following terms: (Central nervous system condition OR Central nervous system disease OR Stroke OR Multiple sclerosis OR Parkinson^*^ disease OR Incomplete spinal cord injury OR Alzheimer^*^ disease) AND (Exercise OR Training OR Physical activity OR Rehabilitation) AND [(Walking OR Gait OR Locomotor) AND (Speed OR Velocity OR Economy OR Expenditure OR Energy or Oxygen)]. All published peer-reviewed articles written in English were retrieved. In addition, reference lists of the retrieved studies were also reviewed. All articles identified were screened by two researchers by reading the title and abstract and evaluated against the eligibility criteria mentioned in the subsequent section.

### 2.2. Inclusion and exclusion criteria

#### 2.2.1. Inclusion criteria

The inclusion criteria were as follows: (1) Participants with a diagnosis of chronic neurological diseases, such as stroke, MS, PD, SCI, and AD. Participants were able to walk alone or with appropriate assistance. (2) Included studies were longitudinal interventional studies, whether randomized controlled trials (RCT), non-randomized controlled trials (N-RCT). (3) Intervention group was based on exercise training, which lasted at least 4 weeks. (4) Walking at self-selected speed (SSS) or absolute speed tested walking economy and standardized for weight or speed. (5) Articles are written in English.

#### 2.2.2. Exclusion criteria

The inclusion criteria were as follows: (1) We excluded studies on exercise combined with other non-physical training on intervention, such as the combination of electrical stimulation, virtual reality, and robot-assisted training. (2) Conference abstracts and posters were excluded.

### 2.3. Data extraction and quality assessment

The search results were downloaded and imported into EndNote software. Duplicates were removed, as well as filter titles, abstracts, and full-text articles. Two authors independently screened the titles, abstracts, and full-text articles. A third author was consulted if there was any discrepancy between the results obtained by the two authors to achieve consensus. The data were independently extracted by two researchers: extraction study design; participant characteristics; intervention description; and oxygen uptake outcome indicators. For the selection of WE, if some studies performed multiple speed measurements simultaneously, for example, 80, 100, and 120% self-selected speed, we included only the 100% group of selected speeds that were most comfortable and closest to life for the participants to ensure homogeneity of results. The article's corresponding author was contacted to clarify or obtain incomplete or missing data.

The methodological quality of the studies was assessed using the original PEDro scale (34). This scale has 11 entries that can be used to assess the methodological quality of physiotherapy. Overall, PEDro has been found to be a valid measure of the methodological quality of clinical trials (35). The evaluation criteria were as follows: eligibility criteria, randomization, concealed allocation, baseline equivalence, blinding of participants, blinding of instructors, blinding of assessors, retention rate of 85%, missing data management (intent-to-treat analysis), between-group analysis, and measures of variability. If the aforementioned information was clear in the study, 1 point was awarded; if not, 0 points were awarded. The maximum score for each study was 11 points. According to the scores, the quality of these studies was divided into four grades: excellent (>9 points), good (6 to 8 points), fair (4 to 5 points), and poor (<4 points) quality.

### 2.4. Exercise definition

The type of exercises was classified into five categories according to the following definitions: (1) ET is defined by the ACSM guideline as a continuous and rhythmic exercise sustained for a period that requires a substantial activation of large skeletal muscles (36), such as treadmill walking or running, and stationary cycling training. (2) RT is defined as a few dynamic muscle contractions against external loads, with sufficient progression (36). (3) ERT is defined as training that includes both endurance and resistance exercises. (4) IT involves repeated high-intensity exercise interspersed with periods of active or inactive recovery (37). (5) OTM is defined as being used to target specific functional disorders. In this study, OTM focuses on the participant's gait at a lower intensity and is designed to restore the patient's ability to walk.

### 2.5. Data synthesis and analysis

Statistical analyses were performed using Comprehensive Meta-Analysis, version 3.0 (Englewood, NJ, USA), with the level of statistical significance set at *p* < 0.05. ES values were calculated from the mean and standard deviation data before and after the exercise intervention or between the experimental and control groups. The effect size was calculated using two methods: (I) For controlled trials, we calculated effect size as the change in the mean of the exercise group before and after the intervention minus the change in the mean of the control group, divided by the combined standard deviation before the intervention, and adjusted for sample size. For studies that included a control group and multiple intervention groups, the sample size of the control group was proportionally reduced. (II) For before-and-after controlled clinical studies without a control group, the effect size was calculated as the mean change before and after the intervention divided by the standard deviation before the intervention, which was presented as Hedges' g and 95% confidence interval (CI).

The type of control group and different exercise types were classified. Subgroup analyses were performed based on each classification, which contained studies larger than two articles. We divided the exercise intervention into pre-exercise and control groups compared with exercise according to the study design. In addition, we also addressed the compliance of the included studies with the published Physical Activity Guidelines (PAG) (38). For studies to meet the PAG, the following conditions had to be met: 150 min/week of moderate-intensity exercise or 75 min/week of vigorous exercise, or roughly a combination of moderate and vigorous exercises. For studies that met the PAG, exercise intensity and duration had to be reported. Moderate intensity was defined as maximum heart rate = 55–70%, maximum oxygen uptake = 40–60%, heart rate reserve = 40–60%, or ratings of perceived exertion (RPE) of 11–13 on the Borg scale. Vigorous intensity was defined as the maximum heart rate of >70%, maximum oxygen uptake of >60%, heart rate reserve of >59%, RPE of >13 on the Borg scale (39).

The magnitude of Hedges' g was interpreted using Cohen's (1988) (40) convention as small (0.2–0.5), medium (0.5–0.8), and large (>0.8). We used the I-squared (I^2^) test to assess the statistical heterogeneity of treatment effects between studies, with I^2^ of > 50% considered heterogeneous. Since the participants included in the study were from different groups of diseases. Therefore, the summary results of the hypotheses are based on the random effects model. The effect of the categorical moderators was based on the significance of the Q_B_ statistic. The Q_B_ statistic indicated the statistical significance of the difference between the levels of the moderator variables. The effect of publication bias on the primary meta-analyses was addressed by combining a funnel plot assessment with Duval and Tweedie's trim and fill correction (41). Sensitivity analysis uses an exclusion-by-exclusion approach to observe whether there is a significant change in the outcome results.

## 3. Result

### 3.1. Study characteristics

**Figure 1:**
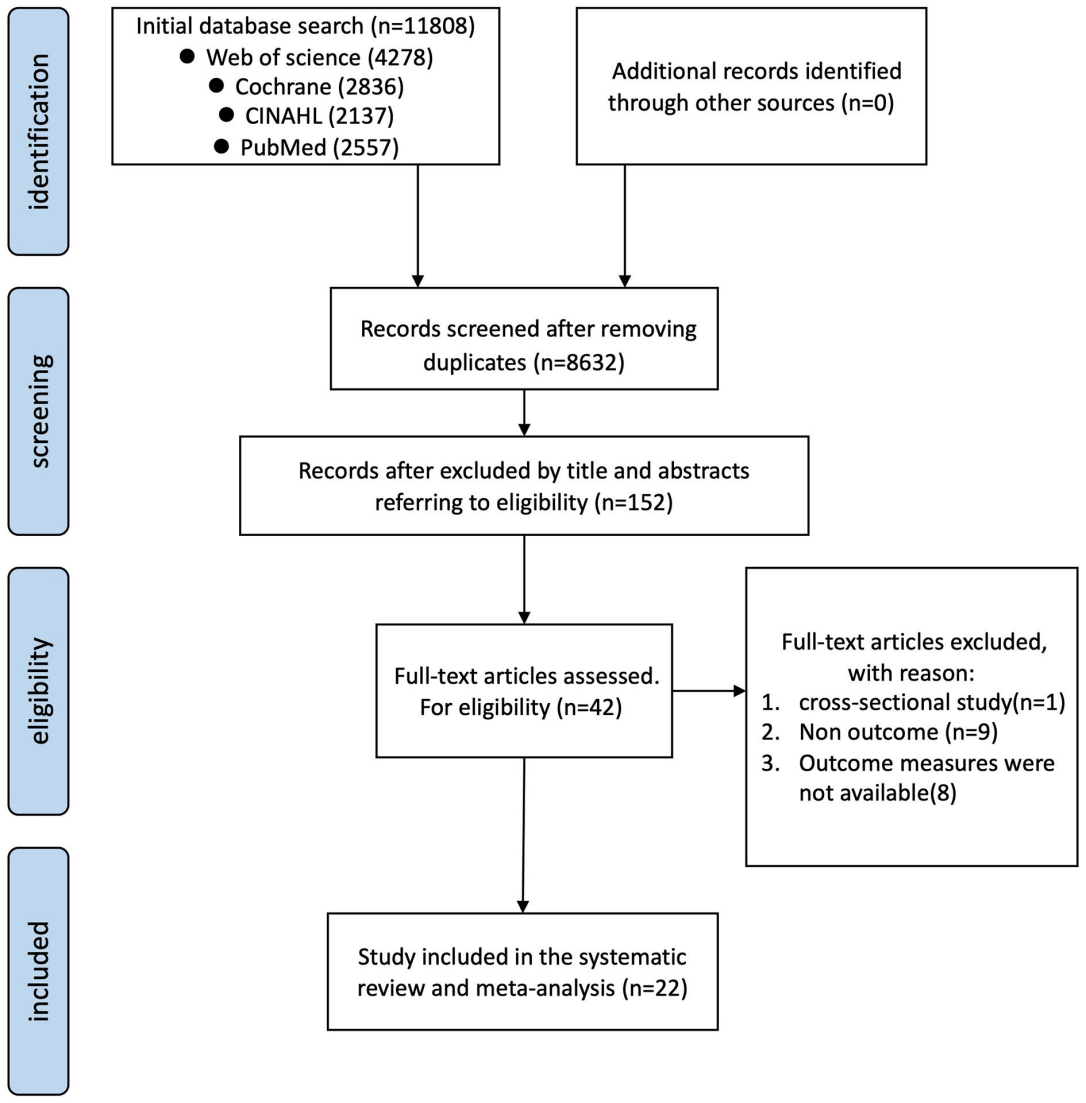
Flowchart of literature search and screening.

Analysis of the four databases yielded 11,808 results. Among others, 8,632 titles and abstracts were screened to remove duplicates. Figure 1 illustrates the number of articles screened and those that met the inclusion criteria. One study was excluded by consensus due to high variability within the participants' group (42). Finally, 22 studies were included in the meta-analysis, yielding a total of 30 interventions (43–64) (Figure 1).

### 3.2. Participants

A total of 612 participants with chronic neurological disorders were included for quantitative analysis, with a mean age of 60.76 ± 8.62. Table 1 presents the participants' characteristics of all included studies. Studies involving patients with stroke (*n* = 13), AD (*n* = 1), PD (*n* = 3), MS (65), and SCI (*n* = 3) were determined. The sample size for each study ranged from 6 to 66. All included studies were supervised, non-home-based clinical intervention trials (Table 1).

**Table 1:** Summary characteristics of the 22 included studies.

**References**	**Participant characteristics**				**Intervention characteristics**				**Walking economy**	

	**Participants**	**Sample** **(male)**	**Age**	**Disease duration (years)**	**Intervention mothed**	**Experimental group intensity**	**Total time**	**Frequent**	**Unit**	**Speed**
Pedrinolla et al. (43)	AD	EG: 16	80 ± 7	NR	EG: Exercise Training	70% HR_max_ 85% 1 RM ^*^12^*^3 groups	90	3 days a week/24 weeks	J/kg/m	Self-selected
		CG: 16	79 ± 6	NR	CG: Cognitive treatment					
Munari et al. (44)	Stroke	EG1: 8(7)	61 ± 5.57	5.2 ± 2.93	EG1: High-intensity treadmill training	Intensity: 85 and 95% VO_2peak_ Recovery: 50% VO_2peak_ walking	50–60	3 days a week/12 weeks	ml/kg/m	Self-selected
		EG2: 7(7)	62 ± 11.27	6.4 ± 3.76	EG2: Low-intensity treadmill training	60% VO2_peak_				
Gollie et al. (45)	SCI	EG: 6	19 to 67	2 to 5	EG: Overground Locomotor Training	NR	90	2 days a week/15 weeks	ml/kg/min	Self-selected
Leddy et al. (46)	Stroke	EG: 12(9)	EG: 55 ± 12	0.478	EG: High-Intensity Dynamic Stepping Training	70–80%HRR	40	4 days a week/10 weeks	ml/kg/m	Self-selected
		CG: 12(8)	CG: 61 ± 10	0.244	CG: Low-intensity physical therapy that includes exercise	30–40%HRR				
Braendvik et al. (48)	MS	EG1: 13(4)	46.6 ± 6.2	8.3 ± 6.4	EG1: Treadmill Training	≤ 70%HR_max_	30	3 days a week/8weeks	ml/kg/min	0.83 m/s
		EG2: 15(5)	49.1 ± 7.4	6.2 ± 6.6	EG2: Progressive Strength Training	Five exercises 80% 1 RM^*^6R^*^2G				
DiPiro et al. (47)	SCI	EG: 10	57.9 ± 9.3	11.1 ± 9.6	EG: Aerobic exercise	40%VO_2_R, increased by 5%. Last week 60–70%VO_2_R	20	2 days a week/6 weeks	ml/kg/min	Self-selected
Boyne et al. (49)	Stroke	EG1: 11(7)	59 ± 9	3.8 ± 2.9	EG1: High-intensity interval training	Start: 30–50%HRR increased by 0.1 mph every 5 seconds Recovery: 40 ± 10 HRR	25	3 days a week/4 weeks	ml/kg/m	Self-selected
		EG2: 5(2)	57 ± 12	6.3 ± 2	EG2: Continuous aerobic training	45 ± 5% HRR, Two weeks later: 50 ± 5% HRR				
Awad et al. (50)	Stroke	EG1: 17(43%)	55.3 ± 5.8	1.73 ± 2.47	EG1: Selected speeds walking	NR	36	3 days a week/12 weeks	ml/kg/m	Comfortable
		EG2: 16(44%)	63.25 ± 5.4	2.68 ± 2.27	EG2: Fast speeds walking	NR				
Holleran et al. (51)	Stroke	EG1: 6	55 ± 8.2	2.92 ± 1.75	EG1: High-intensity locomotor training	70–80% HRR	40	12 sessions/4–5 weeks	ml/kg/m	Self-selected
		EG2: 6			EG2: Low-intensity locomotor training	30–40% HRR				
Fernández-Del-Olmo et al. (52)	PD	EG1: 11(6)	59.45 ± 11.32	4.82 ± 3.28	EG1: Treadmill training	NR	25-45	3 days a week/5 weeks	ml/kg/m	Self-selected
		EG2: 11(6)	58 ± 9.38	4.95 ± 2.59	EG2: Overground training	NR				
Kressler et al. (53)	SCI	EG1: 15	NR	NR	EG1: Overground training	≤ 13 borge	NR	12 weeks	ml/kg/m	Self-selected
		EG2: 17	NR	NR	EG2: Treadmill training	≤ 13 borge				
Ivar Gjellesvik et al. (54)	Stroke	EG: 8(4)	48.9 ± 10.6	7.2 ± 7.5	EG: High Aerobic Intensity Interval Treadmill Walking	85 and 95% VO_2peak_ Interval: 50% HR_max_ walking	90	2 days a week/12–15 weeks	ml/kg/min	0.83 m/s
Hill et al. (55)	Stroke	EG: 11(6)	22 to 61	0.8 to 21	EG: Maximal Strength Training	85–95% 1 RM^*^4^*^4	NR	3 days a week/8 weeks	ml/kg/min	0.83m/s (*n =* 9) 0.75m/s (*n =* 3)
Schenkman et al. (64)	PD	EG1: 41	63.4 ± 11.2	4.9 ± 3.7	EG1: Aerobic exercise	65–80% HR_max_	45–50	3 days a week/64 weeks	ml/kg/min	0.36–1.79 m/s
		EG2: 39	64.5 ± 10	3.9 ± 4.2	EG2: Flexibility/balance/function exercise	NR				
		CG: 41	66.3 ± 10.1	4.5 ± 3.8	CG: Home-based exercise	NR				
Moore et al. (56)	Stroke	EG: 20(14)	50 ± 15	1.08 ± 0.67	EG1: Immediate LT	80–85% HR_max_	NR	2–5 days a week/4 weeks	ml/kg/km	fastest-possible velocity
					EG2: Delayed LT					
Pelosin et al. (57)	PD	EG: 10	69 ± 5.08	7.8 ± 2.14	EG: Treadmill training	Start: 2 km/h, increased by 0.5 km/h 3 days	30	3days a week/4 weeks	ml/kg/min	0.56, 0.69, 0.83, 0.97, 1.11, and 1.25 m/s
Lee et al. (58)	Stroke	EG1: 12(8)	62.6 ± 9.3	3.68 ± 5.33	EG1: Progressive Strength Training	1 Week: 50% 1 RM 2 Week: 80% 1 RM With a 3% increase	60	3 days a week/10–12 weeks	ml/kg/m	Self-selected
		EG2: 12(6)	67.2 ± 10.6	4.52 ± 0.18	EG2: Aerobic Cycle Training	40 rev/min 50–70% HR_max_				
		EG3: 12(8)	60.5 ± 10.6	5.27 ± 0.88	EG3: Aerobic cycling plus Progressive Strength	Ditto				
		CG: 12(6)	65.3 ± 6	5.48 ± 3.53	CG: Sham Exercise	N/A				
Newman et al. (59)	MS	EG: 15	53.6 ± 8.67	17.3 ± 8.3	EG: Aerobic treadmill training	55–85% HR_max_	30	12 session/4 weeks	ml/kg/m	Self-selected
Mead et al. (60)	Stroke	EG: 32(18)	72 ± 10.4	0.15 to 0.79	EG: Progressive endurance and resistance training	13–16 on borge	75	3 days a week/12 weeks	ml/kg/m	Self-selected
		CG: 34(18)	71.7 ± 9.6	0.25 to 0.66	CG: Relaxation intervention	N/A				
Macko et al. (61)	Stroke	EG: 32(22)	63 ± 10	2.91 ± 2.42	EG: Treadmill Exercise	60–70% HRR	40	3 days a week/24 weeks	ml/kg/min	0.22 m/s
		CG: 29(21)	64 ± 8	3.25 ± 4.92	CG: Low-intensity conventional therapy that includes exercise	30–40% HRR				
Macko et al. (62)	Stroke	EG: 23(19)	67 ± 8	>6 months	EG: Treadmill training	60% HRR	40	3 days a week/24 weeks	ml/kg/min	0.22 m/s
Macko et al. (63)	Stroke	EG: 9	67 ± 2.28	3 ± 0.8	EG: Treadmill Aerobic Exercise Training	50–60% HRR	40	3 days a week/24 weeks	ml/kg/min	0.22 m/s

AD, Alzheimer's disease; MS, multiple sclerosis; PD, Parkinson's disease; SCI, spinal cord injury EG, experimental group; CG, control group; NR, not reported; HR_max_, maximum heart rate; HRR, heart rate reserve; VO_2_R, VO_2_ reserve; VO_2peak_, peak oxygen consumption; RM, repetition maximum.

### 3.3. Interventions

Table 1 shows the exercise details for each study. Among the studies included in the meta-analysis, the interventions recorded included IT (*n* = 3), RT (*n* = 3), ET (*n* = 11), ERT (*n* = 3), and OIMT (*n* = 11). The length of intervention ranged from 4 to 64 weeks. The intervention groups in all experiments were based on supervised, non-home exercise. The control group in the six RCT studies used cognitive rehabilitation or usual care (43, 46, 58, 60, 61, 64). Four RCT studies used a usual care control group that contained exercise intervention (46). Table 1 shows the specific intervention intensity and details.

Adverse events were reported in four of the 22 studies (46, 48, 60, 64). They included non-injurious falls, joint pain, and abrasions, but no serious adverse time was recorded. Moreover, these effects were not significantly different between the control and experimental groups. One study reported a fall that occurred outside of the session.

### 3.4. Measurements

The WE measurement primarily involved relative intensity (*n* = 14) and absolute intensity (*n* = 8). All incorporated energy costs or WE were collected using oxygen uptake data obtained directly from indirect thermometry and further processed using body weight or speed. WE measurements under relative intensity measurements were based on self-selected or subject-perceived comfortable speeds, and WE under absolute intensity measurements was in the range of 0.22–1.25 m/s. Most studies were allowed to allow participants to use handrails while walking, or to use other assistance, with one study using 40% weight support in the intervention (56).

### 3.5. Quality assessment

The mean methodological quality score of the 22 included studies was 6.62 ± 1.46. Most studies were of moderate quality, and no studies were rated as low quality (<4). Because most studies used a before-and-after control design, a significant portion of the sample could not meet the requirements for concealed allocation and blinding. No studies were excluded because of methodological quality (Table 2).

**Table 2:** Physiotherapy evidence database (PEDro) scores of the 22 included studies.

**ID**	**References**	**#1**	**#2**	**#3**	**#4**	**#5**	**#6**	**#7**	**#8**	**#9**	**#10**	**#11**	**Score**
1	Pedrinolla et al. (43)	1	1	0	1	0	0	0	1	1	1	1	7
2	Munari et al. (44)	1	1	1	0	0	0	0	1	1	1	1	7
3	Gollie et al. (45)	1	0	0	0	0	0	0	1	1	1	1	5
4	Braendvik et al. (48)	1	1	0	1	0	0	0	0	1	1	1	6
5	DiPiro et al. (47)	1	0	0	0	0	0	0	1	1	1	1	5
6	Leddy et al. (46)	1	1	1	1	0	0	1	0	1	1	1	7
7	Boyne et al. (49)	1	1	1	1	0	0	0	0	1	1	1	7
8	Awad et al. (50)	1	1	0	1	0	1	0	1	1	1	1	8
9	Holleran et al. (51)	1	1	0	1	0	0	0	1	1	1	1	7
10	Fernandez et al. (52)	1	1	0	1	0	0	0	1	1	1	1	7
11	Kressler et al. (53)	1	1	0	1	0	0	0	1	1	1	1	7
12	Ivar Gjellesvik et al. (54)	1	0	0	0	0	0	0	1	1	1	1	5
13	Schenkman et al. (64)	1	1	1	1	0	1	0	1	1	1	1	9
14	Hill et al. (55)	1	0	0	0	0	0	0	1	1	1	1	5
15	Moore et al. (56)	1	1	0	1	1	1	0	1	1	1	1	9
16	Pelosin et al. (57)	1	0	0	0	0	0	0	1	1	1	1	5
17	Lee et al. (58)	1	1	1	1	0	0	0	1	1	1	1	8
18	Newman et al. (59)	1	0	0	0	0	0	1	0	1	1	1	5
19	Mead et al. (60)	1	1	1	1	0	0	0	1	1	1	1	8
20	Macko et al. (61)	1	1	1	1	0	0	1	1	1	1	1	9
21	Macko et al. (62)	1	0	0	0	0	0	0	1	1	1	1	5
22	Macko et al. (63)	1	0	0	0	0	0	0	1	1	1	1	5

#1, eligibility; #2, randomized allocation; #3, concealed allocation; #4, similarity between groups at baseline; #5, Blinding of subjects; #6, Blinding of therapists; #7, blinding of assessors; #8, outcome measures obtained from at least 85% of initially allocated subjects; #9, all received treatment or key outcome was analyzed by “intention-to-treat”; #10, between-group statistical comparisons; #11, both point and variability measures provided.

### 3.6. Meta-analysis

**Figure 2:**
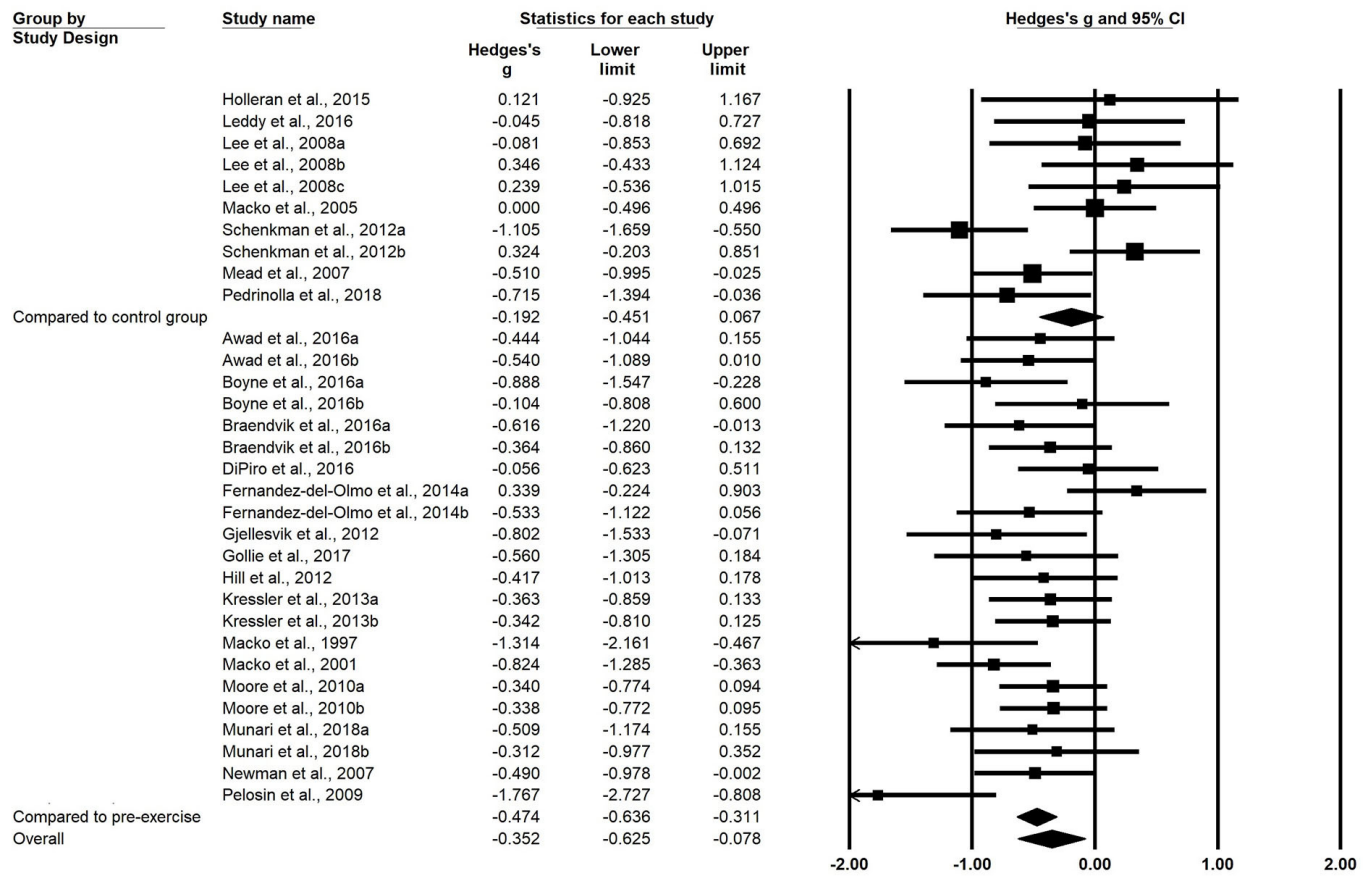
Forest plots of effect sizes and 95% CIs for changes in walking economy after exercise intervention. The superscript numbers refer to different exercise programs assessed in the same study. CI, confidence interval.

A total of 22 studies were included in the meta-analysis. Analysis of the overall pooled results revealed small heterogeneity, with a small but beneficial effect of exercise on WE (g = −0.352, 95% CI: −0.625 to −0.078, *P* = 0.012, I^2^ = 43.301%) (Figure 1). Subgroup analyses were conducted according to the type of intervention in the control group. Pooled results from N-RCTs without controls showed a small effect size improvement in WE after the exercise intervention compared with before intervention (g = −0.474, 95% CI: −0.636 to −0.311, *P* < 0.001, I^2^ = 26.009%). Ten interventions from six RCT studies showed no significant beneficial effect of exercise training on WE compared with controls (g = −0.192, 95% CI: −0.451 to 0.067, *P* = 0.146, I^2^ = 59.349%) (Figure 2).

Subgroup analyses based on the training type revealed that among the included N-RCT studies, nine interventions from seven studies investigating ET (*n* = 7) intervention programs (g = −0.584, 95% CI = −852 to −0.316, *P* < 0.001) and IT (*n* = 3) programs (g = −0.730, 95% CI = −1.169 to −0.292, *P* = 0.001) had moderate effects on WE. OTM (g = −0.361, 95% CI = −0.560 to −0.162, *P* < 0.001) showed a small but beneficial effect on WE.

Three RCTs used ERT as an intervention and five RCTs used ET, none of which resulted in a significant beneficial effect on WE. Other RCTs with exercise interventions were not analyzed in subgroups because the number of aggregates was less than 2 (Table 3). In addition, studies that achieved PAG showed greater improvement in WE compared with pre-exercise, but there was no significant difference between studies that achieved and did not achieve it. In addition, studies that achieved PAG did not show improvement in WE compared with controls.

**Table 3:** Summary results of subgroup analysis for different control group types and exercise type.

**Study design**	**Types of exercise**	**Intervention number**	**Hedges' g**	**Lower limit**	**Upper limit**	***P***	**I^2^**	**Q_B_ (df)**	***P*–total between**
Compared to	ET	7	−0.584	−0.852	−0.316	**<0.001**	61.61%	3.33(3)	0.343
pre-exercise	IT	3	−0.730	−1.169	−0.292	**0.001**	0		
	OTM	10	−0.361	−0.560	−0.162	**<0.001**	0		
	RT	2	−0.387	−0.836	0.061	0.091	0		
	Meeting PAG	5	−0.686	−1.989	−0.383	**<0.001**	0	2.61(1)	0.106
	Not meeting PAG	18	−0.406	−0.559	−0.253	**<0.001**	28.87%		
Compared to	ERT	3	−0.364	−0.951	0.222	0.223	44.917%	0.055(1)	0.815
control group	ET	5	−0.274	−0.748	0.200	0.257	63.05%		
	Meeting PAG	9	−0.250	−0.581	0.082	0.140	54.269%	NA	NA

ET, endurance training; RT, resistance training; IT, interval training; OTM, other training modalities; ES, effect size; PAG, physical activity guidelines; ERT, endurance combined with resistance training; NA, not applicable; Q_B_, Cochran's Q-statistic for subgroup analysis; df, degrees of freedom. Values in bold indicate the presence of significant results.

### 3.7. Publication bias and sensitivity analysis

**Figure 3:**
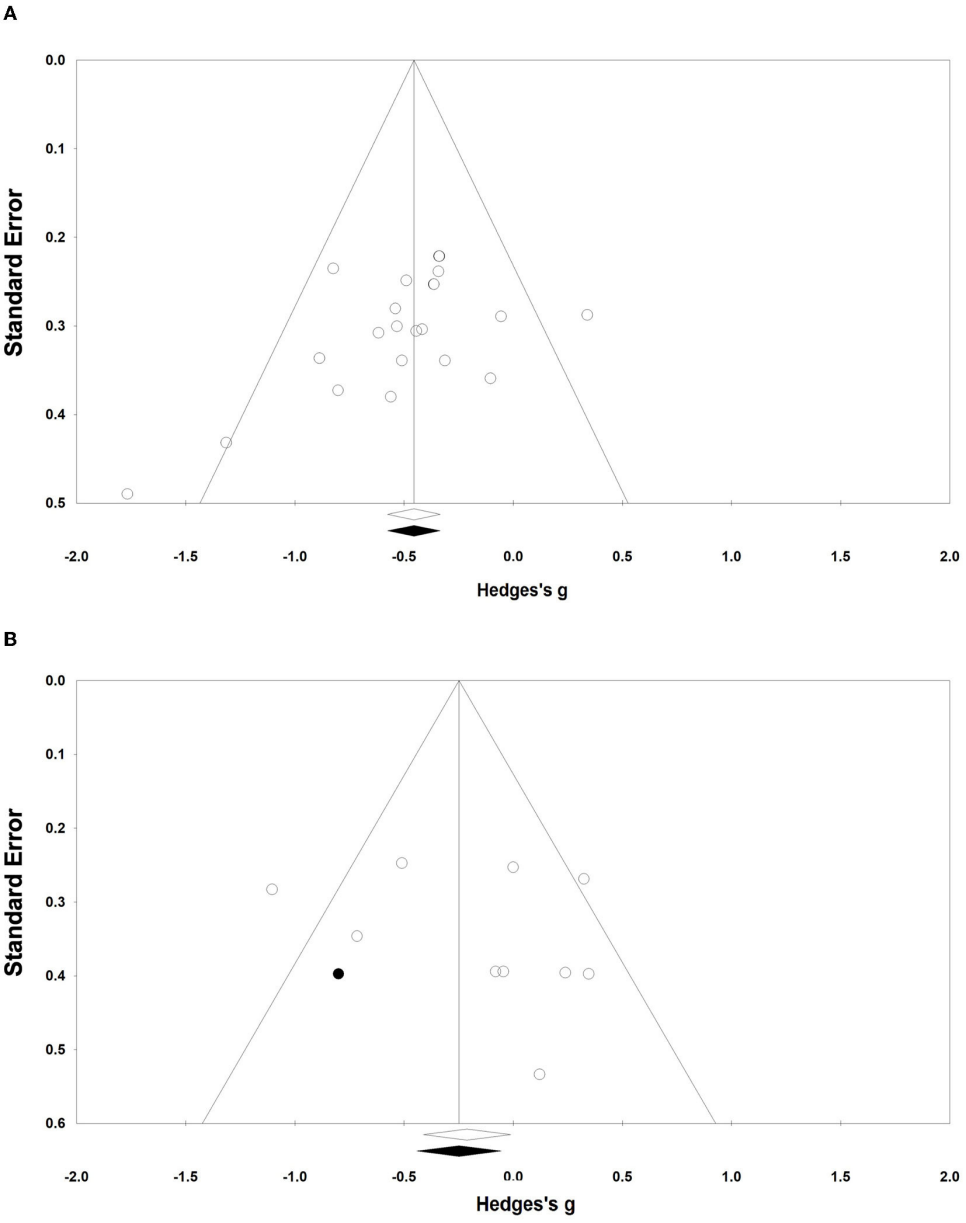
Funnel plots of publication bias. **(A, B)** Funnel plots **(A, B)** represent the publication bias of the N-RCT and the RCT, respectively.

To identify likely publication bias, funnel plots were generated for effect size and standard error. The funnel plots showed that the funnel plots were largely symmetric among the included N-RCT studies. However, studies with disproportionality in RCTs with control groups as a control group were generally located to the right of the variance. One study required adjustments using Duval and Tweedie's trim and fill correction to produce a symmetrical funnel plot around Hedge's g. The correction shifted the overall effect size in the left direction but did not change the main results, although it exhibited a significant trend (Figure 3).

Sensitivity analysis conducted by excluding any of all cohorts from the meta-analysis showed that the estimated effects were within the 95% CI of the mean ES in outcomes. This suggested that the results of the meta-analysis did not significantly change after the removal of any one cohort.

## 4. Discussion

The main objective of this systematic evaluation was to determine the exercise therapies that are used to improve WE in patients with CNCs. We found that exercise improved WE compared with either pre-exercise or non-exercise patients. However, exercise was not more beneficial for WE compared with the control group.

Exercise improves the oxygen cost while walking compared with pre-intervention or no-exercise controls. Several mechanisms may explain the observed results from biomechanics and neuroscience perspectives. In some patients, the improvement in WE is likely related to the biomechanical factors of walking gait because many studies have found an increase in stride length, step length, and a decrease in asymmetrical rows in the affected limb (66). This demonstrates an improvement in the functional capacity of the patient (61). Meanwhile, exercise has been shown to improve the pull reflex in the hamstrings of the lower limbs, and the adaptive responses resulting from these exercises may enhance locomotion, especially the biomechanical efficiency of gait (62). In previous studies, improvements in neurological function in patients with motor stroke were reported, following exercise intervention. Promoting brain plasticity and compensatory activation through high-intensity step training may be a neural mechanism for improving walking gait by stimulating the activation of the subcortical and cortical networks in post-stroke patients (67, 68). For some patients with AD, there is evidence that exercise enhances neuronal and vascular plasticity and improves their pathophysiology (69).

The results of the meta-analysis also showed that higher-intensity exercise did not significantly improve WE compared with the control group. Through a review, we hypothesize that the non-improved outcome may be related to the exercise pattern and the duration of the intervention. Some studies have used usual care in a control group with an exercise intensity of 30–40% heart rate reserve or walking on a treadmill (46, 61). Two of these studies observed significant temporal changes in WE in both the control and intervention groups that lasted for 10 weeks under supervision, that is, the intervention significantly improved WE in the exercise and control groups (46, 61). This result suggests that treadmill-based gait training may improve WE. The previous meta-analysis showed that low-intensity, prolonged treadmill exercise had the greatest benefit on functional impairment in patients with stroke (70). Shulman et al. compared three different intensities of physical activity in patients with PD and found significant differences in fitness and muscle strengthening between the groups, not in gait function. Even lower intensities were superior to higher intensities in some respects (71). It has been suggested that gait training interventions may preferentially increase the oxygen cost of transport instead of enhancing maximal oxygen consumption or lactate thresholds (18). A study by Macko et al. found significantly higher VO_2peak_ in the intervention group compared with the usual care. This is consistent with a previous review that found that high-intensity exercise improves patients' VO_2peak_ (72). Prioritizing WE improvement is an important approach because patients with CNCs have higher energy costs than healthy individuals. In addition, the duration of the intervention was found to affect outcomes. The only study that yielded beneficial effects administered the intervention for up to 16 months. In conclusion, we hypothesize that both higher-intensity and lower-intensity treadmill training can improve WE, but this would take longer. Notably, relaxation and cognitive interventions were used in the control groups of both studies. The results showed a significant improvement in WE in the exercise group compared with the control group (43, 60). This further strengthens our point.

In the study by Lee et al., passive leg cycling resistance training did not significantly improve either VO_2peak_ or WE (58). Therefore, differences in exercise modality were considered. Previous studies have found no significant difference in VO_2peak_ between cycling and treadmill exercise in patients with stroke (73). Moreover, they reported that the choice of exercise modality depended on individual ability and preference. However, the application of cycling to improve gait should be applied with caution because gait is a complex sensorimotor function, and walking and strength-oriented lower extremity therapies are more beneficial to walking ability than cycling (74). A meta-regression based on walking ability in patients with stroke also showed that traditional seated aerobic exercise was unlikely to cause meaningful improvements in walking function (75).

In further analyses, we performed a subgroup analysis to determine the effect of different training types on WE. For ET, pooled results from the N-RCT trials showed a moderate effect on WE. However, it did not have significant benefits compared with conventional rehabilitation (46, 61, 64). About the reviewed results, it does not appear that these two types of training had better effects on WE compared with conventional rehabilitation. Therefore, the effect of ET on WE should be viewed with caution. Compared with other types of exercises, high-intensity IT intervention had the most significant improvement on WE. Unfortunately, there are no higher-quality randomized controlled trials to validate this result. One study found that high-intensity IT had a greater effect on patients' cardiorespiratory fitness than high-intensity exercise alone and sustained aerobic training (10, 12, 76). Due to the increased demand for oxygen during exercise training, the reserves are increased VO_2_, allowing patients to reach higher intensities or greater VO_2_ after training (44). A meta-analysis showed that high-intensity IT induced good adaptations in older adults in terms of cardiorespiratory fitness, body mass, muscle strength, cardiac contractility, mitochondrial citrate, enzyme activity, and lower blood triglyceride and glucose levels (31). Currently, it is not known which type of exercise is more effective in improving WE. However, high-intensity IT has been recognized in many reviews for promoting other physical functions in patients with CNCs (10, 12, 31, 32, 77).

The ERT and OTM interventions showed a small-to-moderate effect on WE. In comparison, previous reviews found that ERT could be the most effective among these interventions in improving cardiopulmonary function in patients with stroke (14). The ASCM guidelines also state that aerobic and resistance exercise is more effective than either form of training in counteracting the adverse effects of a sedentary lifestyle on a healthy cardiovascular system and skeletal muscle function (78). However, the pooled results of the only two RCTs do not confirm that ERT is best for improving WE. Previous systematic evaluations and meta-analyses reported that resistance exercise training had positive effects on overall muscle strength, fatigue, balance, and quality of life in patients with CNCs (11, 79, 80). However, our pooled results only showed a trend of improvement in WE with RT. The confounding effects of our outcome variables make the interpretation difficult. The current evidence only weakly supports the benefit of exercise on WE. In future, continued high-quality randomized controlled trials should be performed to provide more compelling evidence.

Overall, most previous investigations used walking speed, distance, and VO_2peak_ as indicators of cardiorespiratory fitness. This is the first systematic evaluation and meta-analysis that focused on WE and provides valuable ideas for strengthening the cardiorespiratory capacity of patients with CNCs.

### 4.1. Limitations

Some limitations exist in this study. First, a few high-quality RCTs were included. Moreover, most of them were single-arm studies based on before-and-after controls, which may lead to type II errors (e.g., false-negative results) in subgroup analyses. There was some publication bias in the included RCTs, with the pooled results moving in the direction of being more beneficial to WE, although still not constituting significance, which also suggests that there may still be better interventions than traditional rehabilitation. Second, several exploratory preliminary studies with small sample sizes (some below 10 cases) were included in the analysis. Third, while the evidence base was overall of good quality with most studies being of moderate-to-low risk of bias, future research should seek to improve certain points. Of the included studies, most did not use blinding of participants and experimenters based on limitations of the study design. Blinded assignment and assessment of outcomes could limit bias associated with self-report measures in exercise interventions. Finally, the presence of confounding variables in the studies prevented more detailed subgroup analyses, and some results with high heterogeneity could not be interpreted. Therefore, the results of our study should be referred to with caution.

## 5. Conclusion

This systematic review and meta-analysis showed that exercise training improved WE. Notably, the effect of exercise interventions on WE may be the same as usual care appeared to be. Gait-based low-intensity usual care also improved WE. However, it remains to be determined whether there is a more effective means of training that will result in a higher improvement in WE. The prevailing limited evidence suggests that high-intensity IT may be more beneficial for WE compared with other forms of exercise. There is an urgent need for future larger and high-quality studies to find an optimal training modality to improve the cost of walking ability.

## Data availability statement

The original contributions presented in the study are included in the article/supplementary material, further inquiries can be directed to the corresponding author.

## Author contributions

BL, QF, and ZR designed the research. BL and JY conducted the searches and screening process. BL and FH completed the full-text screening. BL, JY, and FH assessed methodological quality. BL, FY, and FH extracted the data, which were checked by JW. BL performed the statistical analysis and interpreted it. BL wrote the manuscript with critical input from ZR and QF. All authors contributed to the article and approved the submitted version.
